# Synthesis of diglycolic acid functionalized core-shell silica coated Fe_3_O_4_ nanomaterials for magnetic extraction of Pb(II) and Cr(VI) ions

**DOI:** 10.1038/s41598-020-67168-2

**Published:** 2020-06-22

**Authors:** Tehreema Nawaz, Sonia Zulfiqar, Muhammad Ilyas Sarwar, Mudassir Iqbal

**Affiliations:** 1 Department of Chemistry, School of Natural Sciences, National University of Sciences and Technology, H-12, Islamabad, 44000 Pakistan; 2 Department of Chemistry, School of Sciences & Engineering, The American University in Cairo, New Cairo, 11835 Egypt; 3 Department of Chemistry, Quaid-i-Azam University, Islamabad, 45320 Pakistan

**Keywords:** Pollution remediation, Magnetic materials

## Abstract

Amine-terminated core-shell silica coated magnetite nanoparticles were functionalized with diglycolic acid for the first time to create acid moiety on the surface of the nanoparticles. The formation of magnetite nanoparticles was scrutinised through XRD, SEM, EDS, TEM, VSM and FTIR spectroscopy. The BET surface area of nano-sorbent was found to be 4.04 m^2^/g with pore size 23.68 nm. These nanomaterials were then utilized to remove the Pb(II) and Cr(VI) ions from their aqueous media and uptake of metal ions was determined by atomic absorption spectroscopy (AAS). A batch adsorption technique was applied to remove both ions at optimised pH and contact time with maximum adsorption efficiency for Pb(II) ions at pH 7 while for Cr(VI) ions at pH 3. Adsorption mechanism was studied using Langmuir and Freundlich isotherms and equilibrium data fitted well for both the isotherms, showing complex nature of adsorption comprising both chemisorption as well as physio-sorption phenomena. The nanosorbents exhibited facile separation by applying external magnetic field due to the ferrimagnetic behaviour with 31.65 emu/g saturation magnetization. These nanosorbents were also found to be used multiple times after regeneration.

## Introduction

Extended release of various heavy metals ions among other toxins in water resources has become a noteworthy issue, due to their intensifying toxic nature, propensity for bioaccumulation and non-biodegradability^1–3^. Lead and chromium are harmful metal ions and released into the ecosystems via various sources i.e., volcanic eruption, non-renewable sources, mineralization, batteries, paints, solid waste disposal. Special glass manufacturing and leather-tanning are two main culprit processes discharging lead ions into environment. Chromium is released via steel fabrication, electroplating, textiles, tanning and wood preservation etc^4–6^. Both chromium and lead are extremely harmful in nature and cause many serious impacts on human beings. Pb(II) is considered amongst the most unsafe heavy metal ions about ecological hazard. This metal is very harmful causing brain damage, encephalopathy; seizures and affects the red blood cells production^7,8^. Rigorous release of Pb(II) can cause heart attack, weight loss and eventually death^9^. Chromium on the other hand is carcinogenic in nature^10^ and found in two valence states: Cr(III) and Cr(VI) where later one is more lethal due to its high adaptability and hyper toxicity. It can also cause detrimental effects on nerves, spinal cords and brain and responsible for physiological damage as well^11,12^. Therefore, effective adsorption of these lethal ions from wastewater is imperative.

A variety of treatment methodologies have already been utilized for elimination of toxic metal ions from contaminated water systems e.g., adsorption, precipitation, ion-exchange method, electrochemical techniques and bioremediation etc^13,14^. But their complex mode of operation, secondary sludge production, high operational cost involved, and reusability issues limit their use to separate the heavy metal ions from water resources. However, in perspective of sustainable development, synthesis of alternative novel environment friendly materials with multiple active sites for adsorption have turned out to be a standout amongst the most critical issues. The development of such kind of materials gives a superior way to eliminate heavy metal ions with more proficiency due to their ease of operation, cost effectiveness and reusability^15^.

Recently, the fabrication and utilization of nanomaterials has gained more importance to adsorb the toxic ions from wastewater. Magnetic nanoparticles (MNPs) considered more competent for this purpose due to their high surface area relative to their volume, tuneable sizes, selective functionalization on the surface and ease of isolation from their aqueous media using external magnetic field. Among MNPs, magnetite (Fe_3_O_4_) nanoparticles are widely studied for their aptitude as nanosorbents for water purification applications. Chemical modification for magnetite nanoparticles provides a wide range of selectivity for heavy metal ions as well as increases their adsorption capability over shorter period^16–19^.

The presence of different functionalities on the surface makes magnetic nanoparticles selectively favourable towards heavy metal ions and prevents the core against agglomeration^20^. In past, different functionalities were introduced on the surface of nanoparticles for selective removal of heavy metal cations or anions from their aqueous media. For example, amine functionalized Fe_3_O_4_ nanoparticles were found promising for the removal of Pb(II), Cu(II), Cd(II) ions from wastewater^21–23^. Similarly, thiol groups were studied against the removal of bivalent Pb, Ag and Hg ions from their aqueous systems^24^. Lately, anhydride group has been used for the immobilization of coexisting amino and acid groups on the surface of magnetic nanocore and used for the removal of Pb(II) ions from their respective media^25^.

There are various reports on the synthesis of silica coated and functionalized Fe_3_O_4_ nanoparticles with different surface groups for the metal uptake studies, more specifically, for the Pb(II) ions targeted in this study. For example, mercaptosuccinic acid (DMSA) modified silica coated Fe_3_O_4_ nanoparticles were found to be effective up to 50.5 mg/g adsorption capacity at room temperature^26^. In another investigation, polypyrrole based surface Fe_3_O_4_ nanoparticles were designed for the effective removal of Pb(II) ions with ~96 percent adsorption capacity^27^. Similarly, graphene oxide is anchored on the surface of magnetite nanoparticles to trap and remove Cr(VI) ions with an uptake capacity of 123.4 mg/g^28^. There are just few reports on the adsorption of Pb(II) and Cr(VI) ions simultaneously by one adsorbent due to different charge of both ions. One such previous report showed the anchoring of amine terminated Fe_3_O_4_ to polyvinyl alcohol/chitosan on polyether sulfone membrane for the effective removal of Pb(II) and Cr(VI) ions^29^. However, the removal capacity varies in each adsorption with the parameters like temperature, concentration and pH etc.

The present study focuses on the synthesis and modification of silica core-shell magnetite nanoparticles (Fe_3_O_4_) with terminal amine groups. These nanoparticles were further functionalized with diglycolic acid to remove the Pb(II) and Cr(VI) ions from their respective aqueous media by varying pH and contact times. Glycolamide based extractants are well known for the heavy metal uptake. In present study these glycolamide based extractants are covalently linked to magnetite nanoparticles. This kind of study has never been reported elsewhere in literature. These extractants were found effective for both metal ions surprisingly, irrespective to the nature of surface moieties, which make them even more encouraging to be used in future for the removal of other heavy metal ions as well. Moreover, their characterization and properties were studied in detail as well as adsorption behaviour was examined using kinetic and isotherm models.

## Materials and methods

### Chemicals

Ferrous sulphate heptahydrate (FeSO_4_.7H_2_O), potassium hydroxide (KOH), tetraethylorthosilicate (TEOS), potassium nitrate (KNO_3_), aminopropyltriethoxysilane (APTES), sodium hydroxide (NaOH) and diglycolic anhydride were purchased from Sigma Aldrich. Phosphoric acid, tetrahydrofuran (THF), acetic acid, ethanol, toluene, lead nitrate, boric acid and potassium dichromate were purchased from other commercial suppliers. All reagents were of analytical grade and were used without further purification. Solvents were freshly dried according to standard protocols for the synthetic work.

### Synthesis of diglygolic acid functionalized nanoparticles (FGA-1)

#### Preparation of Fe_3_O_4_ nanoparticles (F-1)

The magnetite (Fe_3_O_4_) nanoparticles were prepared by a slightly modified previous method named oxidative hydrolysis^26^. Firstly, FeSO_4_.7H_2_O (26 mmol) was dissolved in deionized water (24 mL), followed by the dropwise addition of KOH (40 mmol) and KNO_3_ (3.20 mmol) in 12 mL deionized water under nitrogen purging at 80 °C for 2hrs. This reaction mixture was left for 24hrs at ambient temperature. The nanoparticles formed (designated as F-1) were centrifuged and dried at 60 °C.

#### Silica coating of Fe_3_O_4_ nanoparticles (FS-1)

Sol-Gel process was used to coat Fe_3_O_4_ nanoparticles. For this purpose, initially magnetite nanoparticles (125 mg) were dispersed in ethanol (95 mL) over a sonication bath for 30 min, followed by the slow addition of ammonia solution (7.5 mL) to catalyse the reaction of TEOS (0.25 mL) for silica coating. This solution was further sonicated in an ice bath for 2 hrs. Lastly, silica coated nanoparticles (FS-1) were centrifuged, collected and dried in vacuum oven at 60 °C^30^.

#### Amine functionalized silica coated Fe_3_O_4_ nanoparticles (FSA-1)

For amine functionalization of above-mentioned nanoparticles (100 mg) were spread in toluene (100 mL), then APTES (3 mL) was added. The reaction mixture was refluxed for 24 hrs under nitrogen atmosphere. These amine functionalized nanoparticles (FSA-1) were centrifuged, washed and vacuum dried at 60 °C. A similar method for amine functionalization has been reported elsewhere^31,32^.

#### Diglycolic acid functionalized Fe_3_O_4_ nanoparticles (FGA-1)

**Figure 1:**
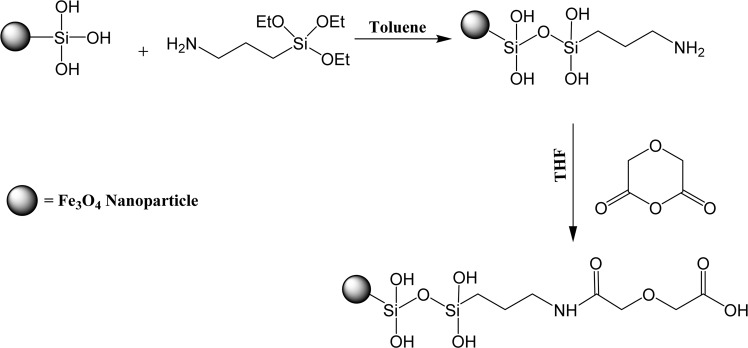
Reaction scheme for synthesis of FGA-1.

The reaction between amine functionalized nanoparticles and diglygolic anhydride was carried out in THF. FSA-1 nanoparticles (100 mg) were dispersed in THF (50 mL) for 15 min, then diglycolic anhydride (200 mg) was added at ambient temperature and reaction mixture was agitated for 48 hrs. Finally, acid functionalized nanoparticles were centrifuged 3 to 4 times using THF and then dried in vacuum oven (named as FGA-1). The reaction scheme for synthesis of FGA-1 is depicted in Fig. 1.

### Adsorption and desorption measurements

#### Adsorption efficiency

The adsorption of both ions was performed by batch equilibrium technique using 10 mg fixed amount of adsorbent against varying concentration of adsorbate solution, pH of aqueous phase and contact time. The adsorption takes place by agitating the adsorbent in the solution at room temperature. After which, adsorbent was recovered using external magnetic field and rest of the solution was analysed by atomic adsorption spectroscopy (AAS) to determine any remaining metal ions concentration. The percentage adsorption and equilibrium adsorption (q_e_) were calculated by the formulae as given below.

1 $$ \% \,Removal=\frac{{C}_{i}-{C}_{e}}{{C}_{i}}\ast 100$$

2 $${q}_{e}=\frac{({C}_{i}-{C}_{e})V}{M}$$

#### Desorption of metal ions

Two different media were used to leach out Pb(II) and Cr(VI) ions, since they are quite opposite in their nature; Pb(II) is a divalent cation while Cr(VI) is a hexavalent anion. To leach out Pb(II) ions, an acidic medium was used having 0.5 M HCl solution and washing the Pb(II) loaded adsorbent for consecutive 4 cycles for the regeneration of adsorbent while for Cr(VI) ions leaching, basic solution of 0.5 M NaOH was used to wash adsorbents for consecutive 4 cycles. The adsorbent was recovered from the supernatant in presence of external magnetic field and the supernatant was analysed for desorption of ions using the following equation.

3 $${q}_{d}=\frac{{c}_{d}v}{m}$$

The desorption efficiency can be determined by the following equation.

4 $$\eta =\frac{{q}_{d}}{{q}_{e}}\ast 100$$

Where C_d_ reflects the ionic concentration present after desorption, V is the volume of acidic/basic solution used and q_e_ is the number of adsorbed species.

## Characterization

The formation of nanoparticles and surface functionalization was monitored by PLATINUM-ATR ALPHA (BRUKER) FTIR in the range of 4000–500 cm^−1^. The crystalline nature of samples was determined by X-ray diffraction technique with an X-ray Diffractometer D8 Advanced Davinci by Bruker having Cu k*α* radiation (1.54 Å) source, operated at 40 KV voltage and 30 mA currents. Morphology and particle size of the adsorbents were determined by scanning electron microscopy (SEM, MIRA3 TESCAN, operating at 10 KV) and transmission electron microscopy (TEM, JEOL, JEM-2100). Energy dispersive X-ray spectroscopy (EDS), a technique coupled with SEM, was used for elemental analysis of the nanomaterials. Zeta potential of the sample was measured using Malvern Panalytical zeta potential analyser. The porosity and Brunauer–Emmett–Teller (BET) surface areas of FSA-1 and FGA-1 samples were obtained using Micromeritics ASAP 2020 physisorption analyzer from N_2_ adsorption isotherms at 77 K. Magnetic measurements were carried out by vibrating sample magnetometer (The Lake Shore VSM 7407, operating at −10 kOe to 10 kOe). The concentration of both ions remained after adsorption was measured by atomic absorption spectrometer (Shimadzu AA-670).

## Results and discussion

### Structural elucidation and phase identification

**Figure 2:**
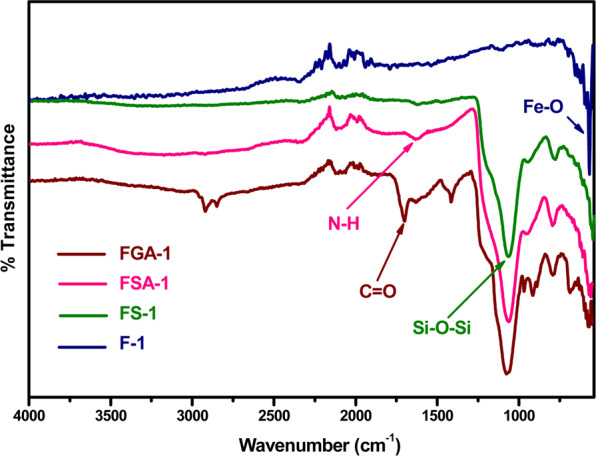
FTIR spectra of Fe_3_O_4_, silica coated, amine and acid functionalized nanoparticles.

The structural elucidation of Fe_3_O_4_, silica coated; their subsequent amine and acid functionalized magnetite nanoparticles were performed by fourier-transform infrared spectroscopy (FT-IR). The technique was used to analyse the nanoparticles and surface groups present on all the prepared samples as shown in Fig. 2. The band at 550 cm^−1^ assigned to the FeO stretching vibration indicating the formation of magnetite nanoparticles having no impurities in the sample. Silica coated Fe_3_O_4_ nanoparticles showed two characteristics vibrations at 790 and 1065 cm^−1^ due to the stretching of Si-O and Si-O-Si bonds respectively. Amine functionalized sample gave additional band at 1629 cm^−1^ due to N-H bending vibration along with Si-O-Si band ascertaining amine modification and a prominent carbonyl stretching vibration at 1697 cm^−1^ along with the amide carbonyl stretching vibration at 1626 cm^−1^ confirming the successful surface functionalization of nanomaterials with acid moieties. Moreover, additional bands can be seen at 690 cm^−1^, 1220 cm^−1^, 1425 cm^−1^ and in the region of 2900–3000 cm^−1^ due to N-H (oop) bending, ether C-O-C stretching, C-N stretching and CH_2_ stretching vibrations. The IR data proved the successful formation, coating and functionalization of the magnetite nanoparticles.

The acid moieties present on the surface of the nanomaterials played a vital role on heavy metal adsorption. Their quantification is usually carried out by spectroscopic and thermal analysis. The data obtained from these methods need careful analysis including baseline correction and deconvolution that can lead to misinterpretations and low reproducibility. However, chemical method that quantifies oxygen-containing functional groups on the surface of nanomaterials is Boehm titration (a back titration). The same titration was performed on the FGA-1 sample and the acidity was calculated as 0.32 mmol/g indicating the presence of the acid functionality on the surface of the nano-sorbents.

The post adsorption IR data was also recorded and presented in Figure S1 (see SI). Both the spectra clearly describe that surface acid functional groups illustrate a major contribution on heavy metal ions adsorption. The pre-adsorbed FGA-1 sample exhibited a band at 1697 cm^−1^ characteristic of carbonyl stretching of the acid moieties present on the surface. The same band was shifted to the lower wave number in the post adsorbed FGA-1 for both metals ions (Figure S1) i.e., 1554 and 1612 cm^−1^ for Pb(II) and Cr(VI) respectively. This observation clearly verified that acid functionalized magnetite nanoparticles interacted with metal ions and hence this nano-sorbent facilitated the removal of heavy metal ions from wastewater.

**Figure 3:**
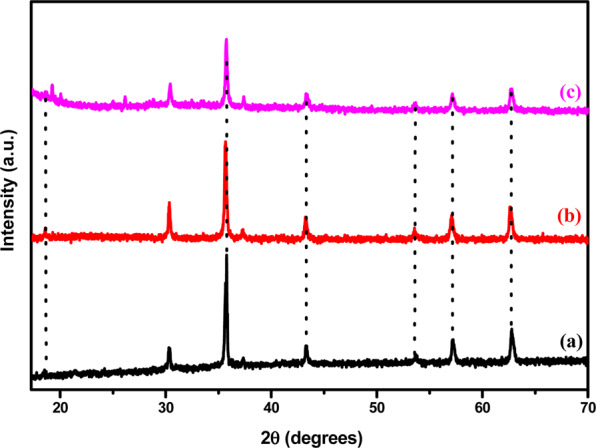
XRD patterns of (**a**) Fe_3_O_4_, (**b**) silica coated and (**c**) acid functionalized nanoparticles.

Crystallinity and phase identification of coated and functionalized magnetite nanoparticles were carried out by X-ray diffraction technique and XRD patterns recorded for 2θ in the range of 25° to 70° on the powdered samples are shown in Fig. 3. All the samples exhibited the same diffraction patterns at 2θ = 30.1°, 35.5°, 37.1°, 43.1°, 53.5°, 57.0° and at 62.6°, corresponding to [311], [222], [400], [422], [511] and [450] hkl values. These observations indicate the single phase materials and the XRD pattern of magnetite crystal structure is in good agreement with the JCPDS 01–075–0033 powder diffraction. It further suggest well retained magnetic cores present in all samples when compared with the initial Fe_3_O_4_ nanoparticles except for the distinct peak appeared at 2θ = 20° corresponding the diffraction peak for silica groups^15^. Moreover, the diffractograms show no peak shifting upon coating and functionalization meaning that the crystalline phase and stability of magnetite nanoparticles persevered. The crystallite size of magnetite nanoparticles was also determined by Scherrer formula using XRD data and the average particle size was 45.08 nm.

### Morphology and elemental composition

**Figure 4:**
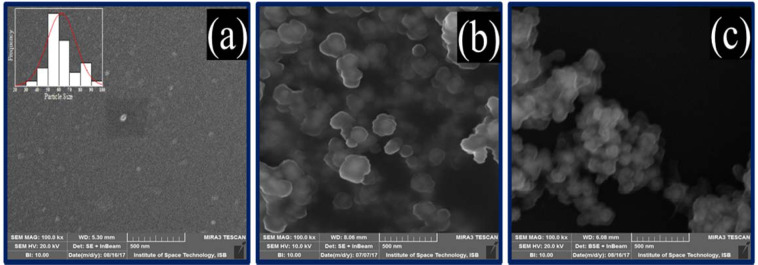
SEM images of (**a**) Fe_3_O_4_, (**b**) silica coated and (**c**) acid functionalized nanoparticles.

Morphological investigations on the powdered samples of magnetite, silica coated and acid functionalized nanoparticles were carried out by scanning electron microscopy and SEM images are presented in Fig. 4. The micrographs show a quasi-spherical shape with particles showing little agglomeration having an average particle size 55.5 ± 5.5 nm (Fig. 4(a)), which is close to the crystallite size obtained from the XRD data. After silica modification, particle size is increased with a cloudy shell-like appearance on the surface providing evidence about the presence of amorphous functionalities around the nanoparticles (Fig. 4(b)). This cloudy shell becomes thicker with increasing number of functionalities on the surface. Figure 4(c) shows more thickened cloudy shell on the surface of acid functionalized nanoparticles without affecting the crystalline phase and stability of magnetite nanoparticles. Additionally, these particles also appeared to be agglomerated due to the nanosized interacting surfaces.

**Figure 5:**
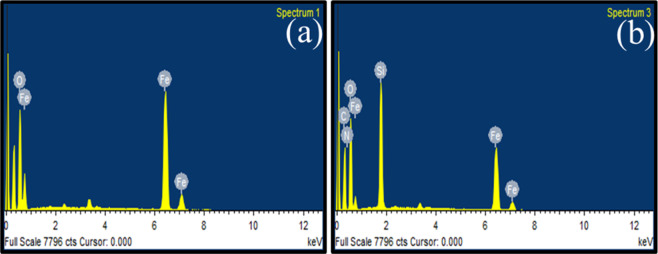
EDS spectra of (**a**) Fe_3_O_4_ and (**b**) acid functionalized nanoparticles.

Energy-dispersive X-ray technique usually coupled with SEM give valuable information regarding the chemical composition of the materials. The elemental composition of magnetite nanoparticles was monitored by recording the EDS spectra as given in Fig. 5. The pristine magnetite nanoparticles gave two peaks for Fe and O elements showing the purity of synthesized nanoparticles (Fig. 5(a)). Figure 5(b) indicated EDS spectrum of diglycolic acid functionalized nanoparticles with additional peaks of carbon, nitrogen and silicon.

In acid functionalized spectrum, more elements are expected relative to pure magnetite nanoparticles due to silica coating and functionalization of surface with amine and acid moieties. Moreover, oxygen percentage also increased in the later spectrum due to the surface bearing one ether (-O-) and two acid groups (-COOH, -NHCO).

**Figure 6:**
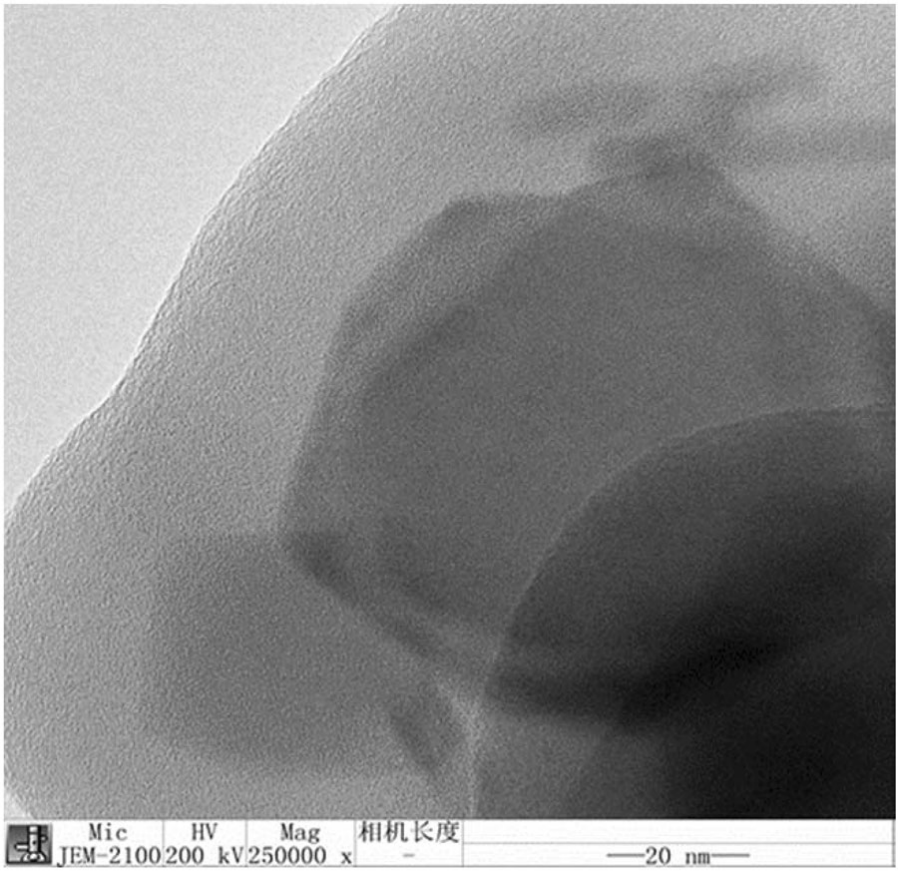
TEM image of acid functionalized nanoparticles.

The internal morphology of diglycolic acid functionalized nanoparticles was also monitored by transmission electron microscopy and the TEM image is presented in Fig. 6. The micrograph depicted the presence of cloudy shell of acid functionalized nanoparticles showing an average particle size of core approximately 60 nm and 20 nm shell around on the surface. Furthermore, it confirmed that functionalization did not affect the crystallinity and stability of magnetic core.

### Magnetic measurements

**Figure 7:**
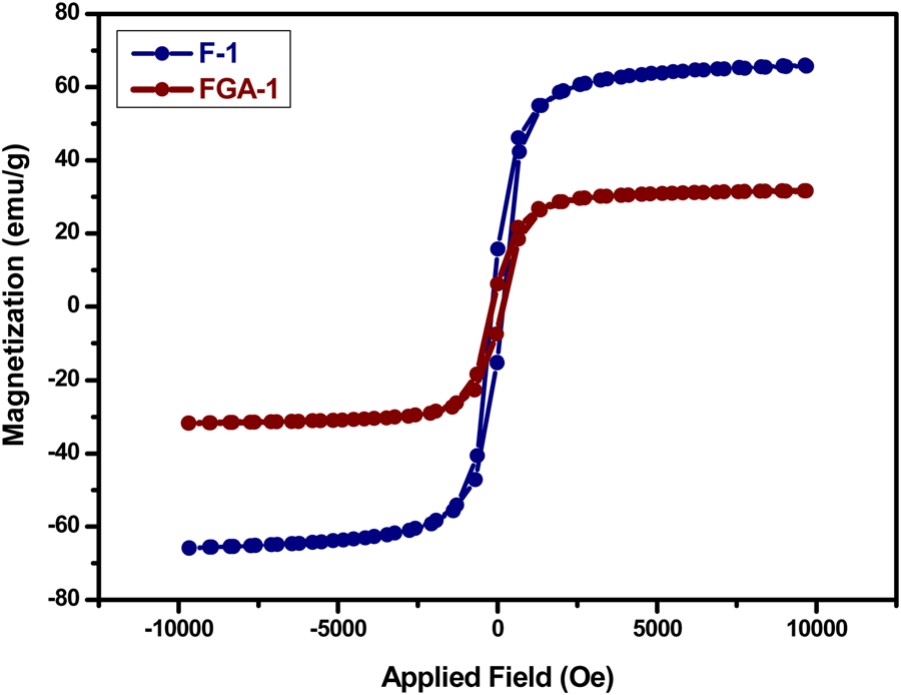
VSM measurements of Fe_3_O_4_ and acid functionalized nanoparticles.

VSM method is used to determine the magnetic properties of nanomaterials based on the change of magnetic field produced by the magnetometer’s vibrating component. The magnetic behaviour and hysteresis loops of pure and acid functionalized magnetite samples as measured by VSM is shown in Fig. 7. The mass saturation magnetization value (M_*s*_) obtained for pristine Fe_3_O_4_ nanoparticles was 65.96 emu/g, which was further reduced to 31.65 emu/g for acid functionalized nanoparticles due to the incorporation of non-magnetic shell (silica/amino/acid) around the surface. These observations are in good agreement with the previous studies that functionalization causes a decrease in saturation magnetization^33–35^. However, these samples showed large values of remanence (M_r_) and coercivity (H_c_) favouring ferromagnetic behaviour (Table 1). The other evidence in favour of ferrimagnetic nature comes from the particle size i.e., more than 20 nm excluding the paramagnetic anticipation in the nanoparticles. These measurements are in line with reported values in the literature^36–38^.

**Table 1:** VSM parameters of Fe_3_O_4_ and acid functionalized nanoparticles.

Sample	M_r_(emu/g)	H_c_ (O_e_)
F-1	15.764	247.25
FGA-1	6.06	194.44

### Zeta potential, surface area and porosity measurements

**Figure 8:**
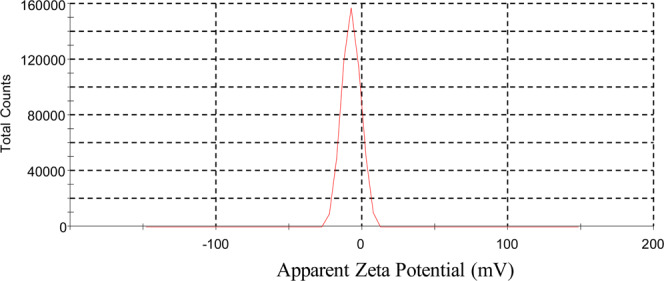
Zeta potential measurement of FGA-1 at 25 °C.

Zeta potential is the measure of potential difference across phase boundaries between solids and liquids. Zeta potential measurement and the surface charge on FGA-1 at pH 7 is given in Fig. 8. The zeta potential is negative (−7.39) as expected due to presence of carboxylate anions on the surface at pH 7. This relates with the adsorption studies carried out on metal ions at various pH values. The surface of nano-sorbent is negatively charged at this pH value due to ionization of carboxylic acid, adsorption of Pb(II) ions is preferred relative to Cr(VI) ions. Slight change for Pb(II) adsorption was observed at higher pH values, however, the adsorption of Cr(VI) was decreased. At lower pH values, the surface is assumed to be protonated facilitating the adsorption of Cr(VI) ions more relative to Pb(II) cations. The same trend has been observed while moving from higher to lower pH values.

**Figure 9:**
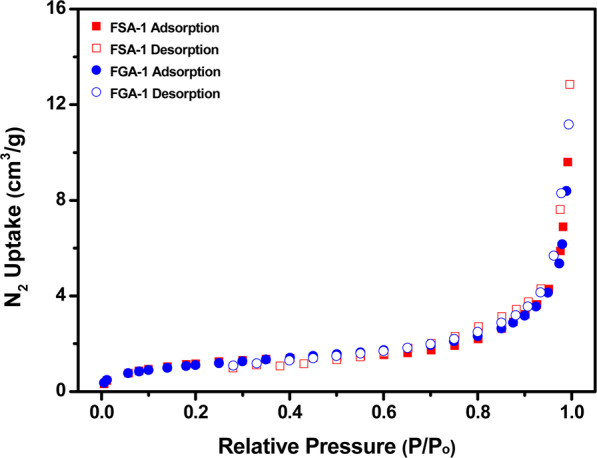
N_2_ gas isotherms of FSA-1 and FGA-1 measured at 77 K.

Moreover, BET measurements on FSA-1 and FGA-1 samples were performed using N_2_ adsorption-desorption isotherms (Fig. 9) to evaluate textural parameters such as surface area, pore volume and pore size of nano-sorbents as described in Table 2. The BET linear plots of FSA-1 and FGA-1 from N_2_ isotherms at 77 K are shown in Figure S2. The obtained parameters showing a mesoporous structure with narrow range of pore size distribution. These values are in favour of electrostatic adsorption phenomena, as surface area of nanoparticles is less due to more size and more functionality on the surface. However, physisorption cannot be neglected completely and seems more favourable in case of Cr(VI) ion.

**Table 2:** Textural parameters of FSA-1 and FGA-1 calculated from N_2_ adsorption isotherms at 77 K.

V	SA_BET_ (m^2^/g)	SA_Langmuir_ (m^2^/g)	Pore Size (nm)	Pore Volume (cm^3^/g)
FSA-1	4.24	6.63	33.00	0.019
FGA-1	4.04	6.09	23.68	0.016

### Metal ions uptake

The synthesized nanosorbents comprising terminal acid moieties on their surfaces can be exploited as excellent chelating sites for heavy metal ions, thus removing metal cations from wastewater and effluents of various industries. In the present study, lead (II) and chromium (VI) were preferred for uptake measurements because these metals ions have excellent coordination with acid groups present on the surface of nanoparticles and the results were evaluated by Langmuir and Freundlich models. To achieve an optimal adsorption, certain parameters (time, pH and adsorbate concentration) were optimised over a constant dosage of adsorbent at room temperature. For each experiment, 10 mg of adsorbent dosage was used as previously optimized ^32^ over the variable range of one parameter to get the maximum adsorption limit and after that the same procedure was repeated with all other parameters. Average values of three runs of each parameter optimized were taken with ± 0.01 standard deviation.

#### Effect of time

**Figure 10:**
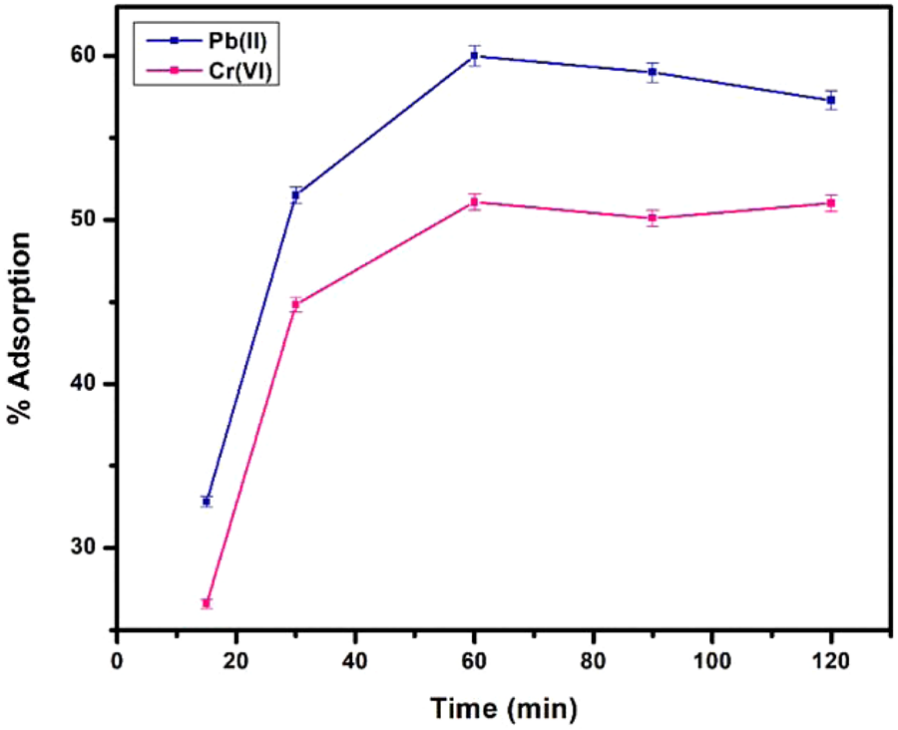
Adsorption of metal ions as a function of time.

Time was the first parameter to be optimised inceptively for both the metal ions. The solution of each ion (10 ppm) was agitated with 10 mg of acid functionalized nanomaterials at neutral pH and room temperature over a variable range of time intervals (15, 30, 60, 90 and 120 min). Figure 10 summarises optimum adsorption with time for both the metal ions up to 60 min at equilibrium point and then it almost level off with further increase in time. However, nanomaterials showed more adsorption for Pb(II) cations than Cr(VI) ions due to negatively charged surface groups, favoured the adsorption of cations and the maximum adsorption for Pb(II) ions were ~60% and ~50% for Cr(VI) ions. The theoretical values of q_e(Theoretical)_ and also agree with the experimental values q_e(Experimental)_ exhibiting that the present adsorption system follows to the pseudo-second-order mechanism and the adsorption rate is controlled by chemical sorption (Table S2 and S4).

Kinetic modelling not only allows estimation of sorption rates but also leads to suitable rate expressions characteristic of possible reaction mechanisms. Therefore, pseudo-first order and pseudo-second order kinetics models were applied on the adsorption data as a function of time for heavy metal removal. The respective kinetics and kinetic parameters of these two models investigated on the adsorption data of both metal ions can be seen from the Figures S3–S6 and Tables S1–S4. Pseudo-first order was found to be inapplicable while pseudo-second order kinetic model was applicable with R^2^ values 0.99 for both the metal ions. In this model, the rate-limiting step is the surface adsorption that involves chemisorption, where the removal from a solution is due to physicochemical interactions between the two phases.

#### Effect of pH

**Figure 11:**
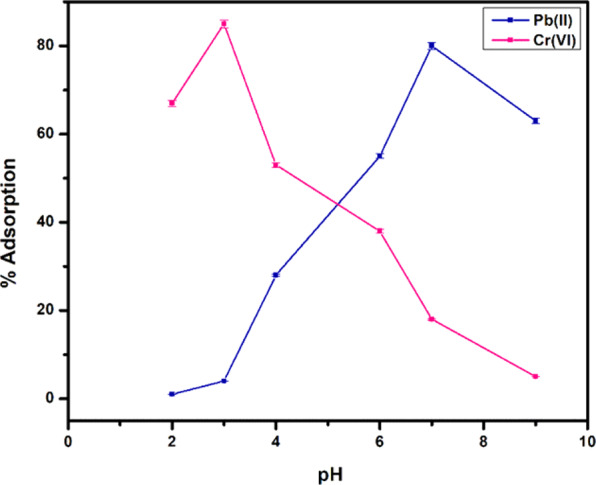
Adsorption of metal ions as a function of pH.

pH has vigorous effect on metal uptake as varying pH values causes a tremendous change for the adsorption of both the metal ions. The effect of pH was optimised at constant adsorbent concentration (10 mg), time (1 h) at room temperature. Since Pb(II) ions are positively charged species and inherently attractive towards the negative charge. So, pH was varied from acidic to basic and the adsorption behaviour is depicted in Fig. 11. The uptake was maximum at neutral pH, tangible evident showing the tendency of active sites to attract positively charged species Pb(II) when these are more likely to be unoccupied. This is because at lower pH values, more protonated surfaces block the binding of Pb(II) cations and reduces the adsorption rate while at higher pH values, concentration of OH^-^ ions competes for Pb(II) cations and reduces the adsorption rate. The maximum pH observed was at 7 pH of the solution and almost negligible at acidic or basic pH values. Cr(VI) showed opposite trend and surprisingly more adsorption was observed at lower pH values i.e. up to ~85 percent adsorption at acidic pH = 3. This is because the more protonated surfaces tends to attract more Cr(VI) ions towards themselves causing the more adsorption of Cr(VI) ions that decreases further increasing the pH value, possibly due to protonation of chromate ions.

#### Effect of adsorbate concentration

**Figure 12:**
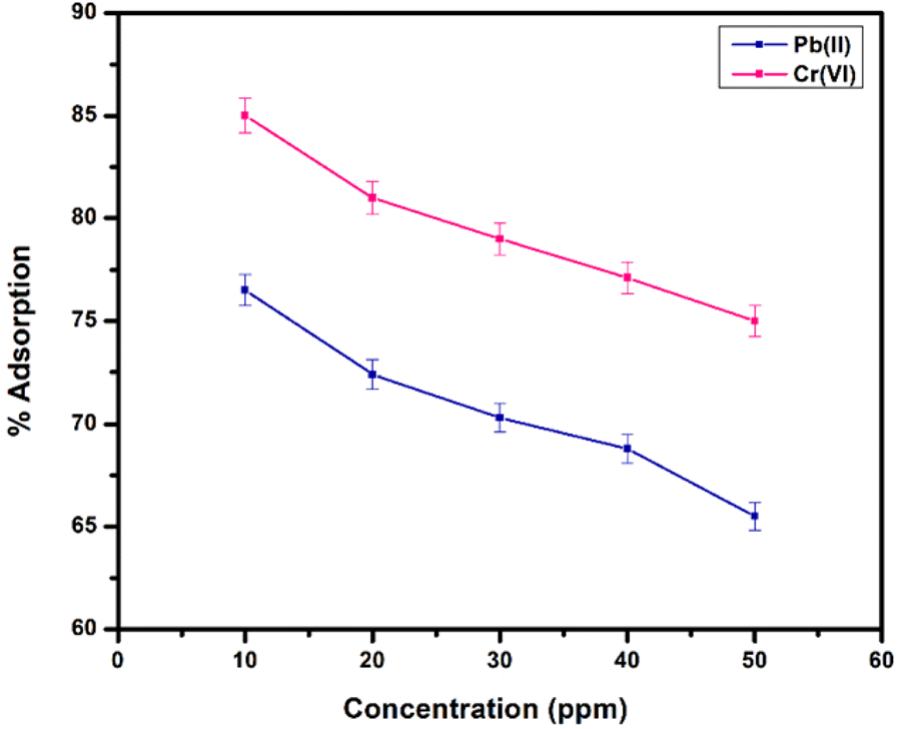
Adsorption of metal ions as a function of adsorbate concentration.

The effect of metal ions concentration was studied to describe the adsorption kinetics at maximum adsorption capacity of the adsorbents. Figure 12 explains the adsorption behaviour at optimised values of time (1 hr), pH = 7 for Pb(II) cations and pH = 3 for Cr(VI) ions at room temperature per 10 mg of acid functionalized magnetite. While the concentration of metal ions was varied from 10–50 ppm and saturating the solution with metal ions have adverse effect on the adsorption rate. Because all the available active sites are already occupied that decreased the tendency to pick up more metal ions in the solutions. However, the maximum adsorption was observed at 10 ppm solution, optimised value over 10 mg of acid functionalized adsorbent’s concentration beyond further increase in concentration was unfavourable for maximum adsorption capacity.

#### Adsorption isotherms

The adsorption mechanism was studied by Dubinin-Raduchkevick, Langmuir and Freundlich isotherm models. The parameters derived from these three models provide much information about the affinity of adsorbent, surface properties and the nature of adsorption. Dubinin-Raduchkevick isotherm model was applied on the adsorption data for both the metal ions and this model was found to be not applicable as can be seen from Figures S7 and S8 and D-R isotherm parameters (Tables S5 and S6).

Langmuir adsorption isotherm states that the adsorption phenomena are monolayered because of the limited number of homogeneous active sites on adsorbent’s surface, where if one site is occupied by a particle, no further adsorption is possible. Langmuir adsorption isotherm’s linear form is given by the following equation.

5 $$\frac{1}{{{\rm{q}}}_{{\rm{e}}}}=\frac{1}{{{\rm{Q}}}_{0}}+\frac{1}{{{\rm{bQ}}}_{0}{{\rm{C}}}_{{\rm{e}}}}$$

where, q_e_ = equilibrium concentration of adsorbed ions, Q_0_ = maximum removal capacity, Ce = equilibrium concentration of adsorbate

**Figure 13:**
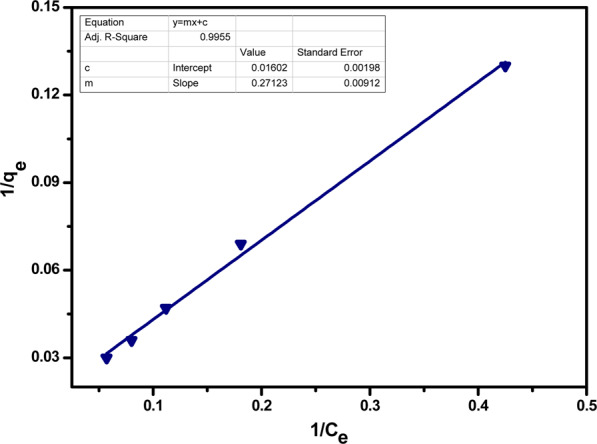
Langmuir isotherm for adsorption of Pb(II) ions by acid functionalized nanoparticles.

**Figure 14:**
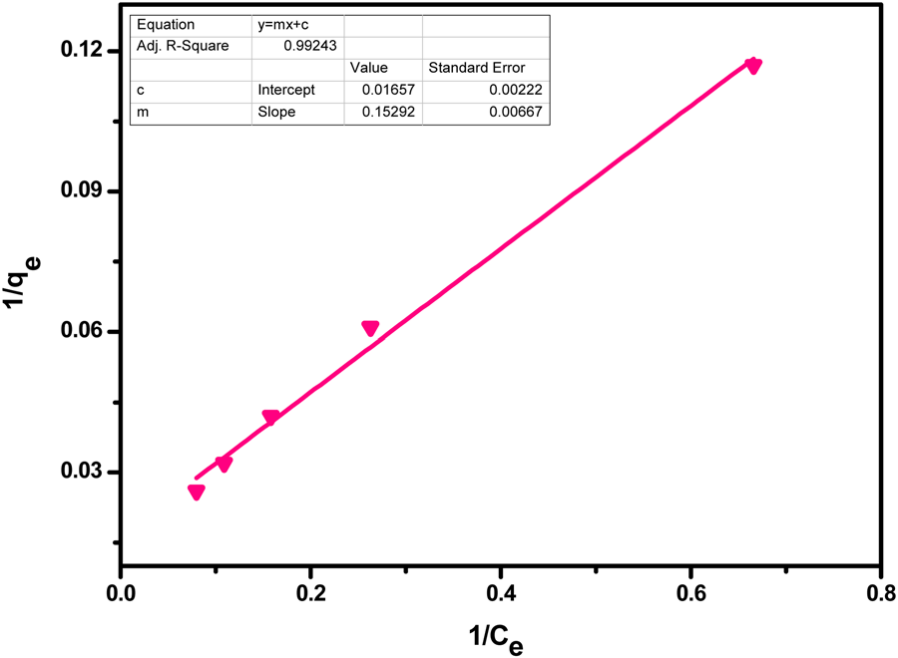
Langmuir isotherm for adsorption of Cr(VI) ions by acid functionalized nanoparticles.

Graphical representation of Langmuir adsorption isotherm for Pb(II) and Cr(VI) adsorption at the surface of acid functionalized nanoparticles was obtained by plotting of 1/C_e_ against 1/q_e_ using the above straight-line equation as shown in Figs. 13 and 14. The parameters of Langmuir adsorption isotherm are given in the Table 3.

**Table 3:** Langmuir isotherm parameters.

Metal ions	Q_o_ (mg/g)	B or K_L_ (L/mg)	R^2^
Pb(II)	62.42	0.59	0.99
Cr(VI)	60.35	0.11	0.99

Where, K_L_ or b shows the increased affinity of the adsorbent for adsorbate ions, Q_o_ is the maximum adsorption capacity of the adsorbent. The value for R^2^ depicts that adsorption is favourable by Langmuir adsorption isotherm. In graphical representation, a dimensionless constant RL is used for the adsorption studies. If RL > 1, adsorption will be unfavourable if RL = 1, adsorption is linear and if 0<RL < 1, adsorption will be favourable and if RL = 0, adsorption will be unfavourable.

6 $${R}_{L}=\frac{1}{1+b{Q}_{O}}$$

The value of R_L_ from the above equation is found to be higher than 0 but less than 1, depicting that adsorption is favourable in both cases. The Langmuir process thermodynamic was ensured by calculating of ΔG values for Pb(II) = ‒1.59 KJmol^−1^ and for Cr(VI) = ‒2.78 KJmol^−1^. These negative values indicated that sorption of both the metal ions is spontaneous and thermodynamically feasible.

Freundlich isotherm is known to describe the non-ideal and reversible adsorption behaviour in favour of the development of multilayers. This observational model is used to describe the heterogeneous surface for non-uniform distribution adsorption affinities. This isotherm says that the occupation of active sites depends upon their strength, as most strong sites will be occupied first. Freundlich adsorption isotherm’s linear form is given as

7 $$\log \,{{\rm{q}}}_{{\rm{e}}}=\,\log \,{{\rm{k}}}_{{\rm{F}}}+\frac{1}{{\rm{n}}}\,\log \,{\rm{Ce}}$$

where, K_F_ = Freundlich constant, n = Heterogeneity factor

**Figure 15:**
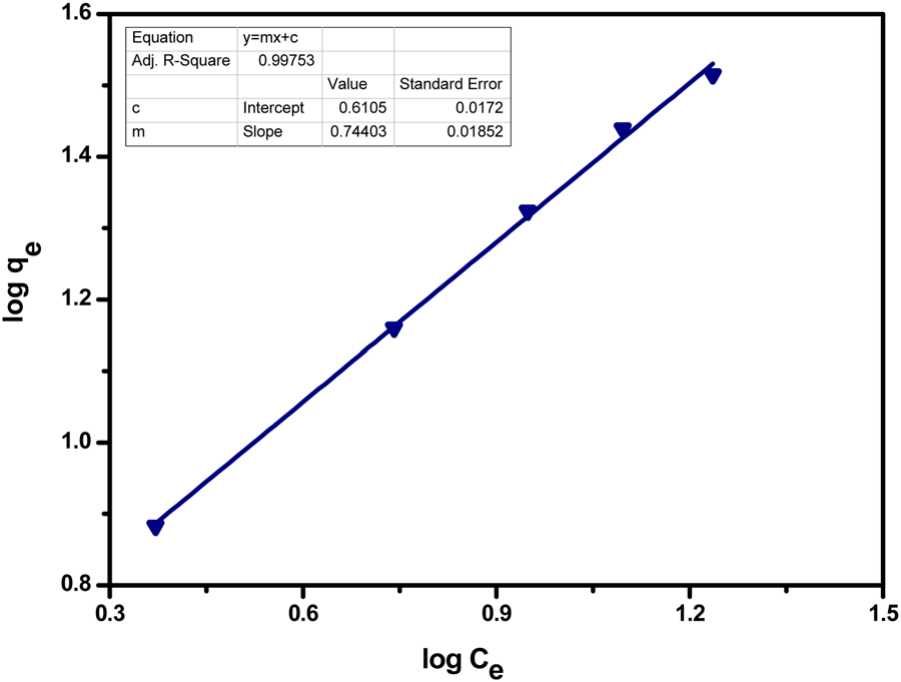
Freundlich isotherm for adsorption of Pb(II) ions by acid functionalized nanoparticles.

**Figure 16:**
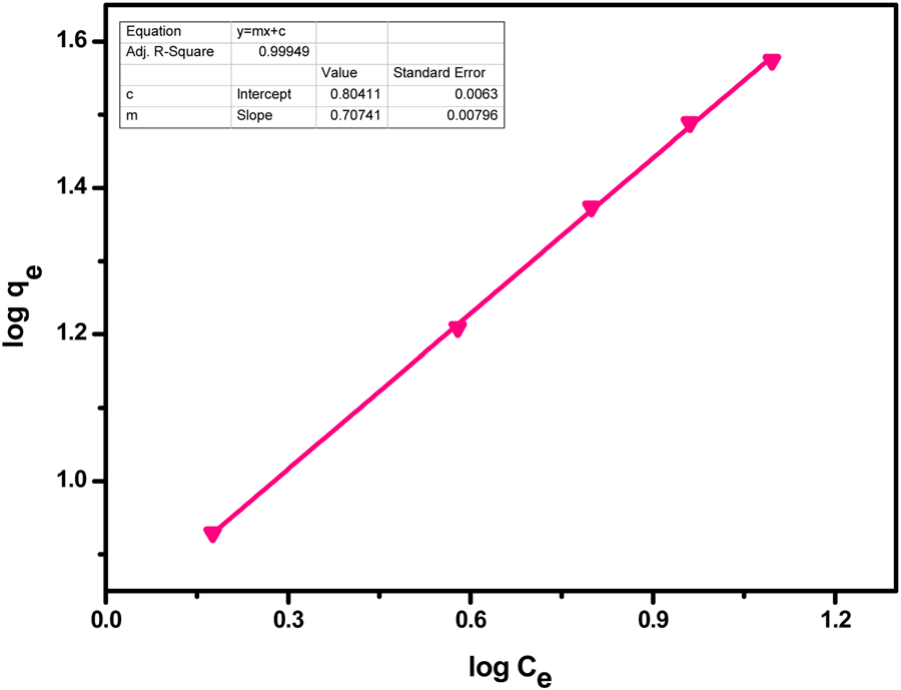
Freundlich isotherm for adsorption of Cr(VI) ions by acid functionalized nanoparticles.

Linear Freundlich isotherm plots are given below in Figs. 15 and 16. The parameters of this isotherm are given in Table 4. The *n* value denotes the degree of nonlinearity between solution concentration and adsorption as follows: if *n* = 1, then adsorption is considered as linear; if *n* < 1, then adsorption is through a chemical process; if *n* > 1, then adsorption is via a physical process. The values of *n* = 1.35 and 1.40 for Pb(II) and Cr(VI) respectively is the most common feature of this model yielding from the distribution of surface sites or any factor that causes a decrease in sorbent-sorbate interaction with increasing density.

**Table 4:** Freundlich isotherm parameters.

Metal ions	K_f_ (mg/g)(L/mg)^1/n^	n	R^2^
Pb(II)	0.61	1.35	0.99
Cr(VI)	0.80	1.40	0.99

Both the models are in favour of adsorption for metal ions with slight preference of Freundlich adsorption isotherm for acid functionalized nanoparticles, as metals can be adsorbed by forming multi-layered surfaces on the nanosorbents due to the presence of heterogeneous active sites. It showed that adsorption process is a complex process comprising two kinds of interactions i.e. electrostatic attraction between adsorbent surfaces and metal ions and chemical bonding between the surface groups and metal ions.

### Adsorbent regeneration

**Figure 17:**
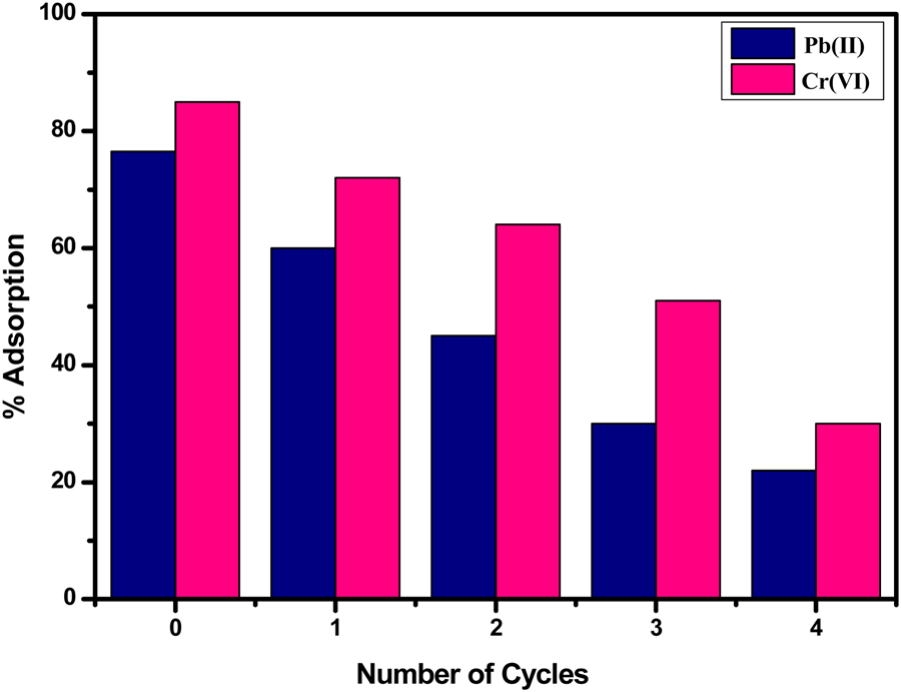
Adsorption capacity of Pb(II) and Cr(VI) ions on regeneration of acid functionalized nanoparticles.

The recycling of the nanosorbents makes them cost-effective in the practical applications. The recovery of nanosorbents was evaluated till four consecutive cycles and after each cycle, adsorption efficiency was decreased for both metal ions. However, still it can be used up to 4–5 cycles to remove these toxic metal ions from wastewater. The reusability results are given in Fig. 17. The possible reduction in adsorption efficiency might be either due to incomplete stripping of metal ions as evident from Langmuir and Freundlich isotherms that adsorption involves both physio-chemical interactions. Another possible reason for decreased efficiency in each cycle might be partial erosion of adsorbent surface in strong acidic or basic media used during regeneration.

## Conclusions

New efficient acid functionalized magnetite nanosorbents were prepared and their adsorption capacities for Pb(II) and Cr(VI) ions were investigated from their respective aqueous media through a batch technique. The structure evaluation confirmed the presence of acid moieties on the surface (0.32 mmol/g) as determined by Boehm titration and thick cloudy shell around the magnetic core. The adsorption efficiency of these nanosorbents for both metal ions was studied at optimised parameters giving high adsorption capacity for Pb (II) cations (62.42 mg/g) and lower adsorption capacity (60.35 mg/g) for Cr (VI) anions. The interactions between adsorbents and adsorbates are both time and pH dependent. The data also indicate that these nanosorbents can rationally be applied at both neutral and acidic environments. The adsorption data is in line with the Langmuir and Freundlich adsorption isotherms, however, more favouring Freundlich isotherms depicting both physisorption and chemisorption phenomena are operative for the uptake of metal ions. These nanomaterials displayed ferromagnetic behaviour with 31.65 emu/g saturation magnetization, which could easily be isolated by EMF. High adsorption efficiency as well as reusability till 4 to 5 cycles was found for these nanosorbents.

## Supplementary information

Supplementary Information.
