# Supplementary material for: Synthesis of diglycolic acid functionalized core-shell silica coated Fe3O4 nanomaterials for magnetic extraction of Pb(II) and Cr(VI) ions
Source: Sci Rep. 2020 Jun 22;10:10076. doi: 10.1038/s41598-020-67168-2 (PMC7308298; doi:10.1038/s41598-020-67168-2)
Supplement: Supplementary file 1 — Supplementary Information. [file 41598_2020_67168_MOESM1_ESM.pdf]

Supplementary Information

Synthesis of diglycolic acid functionalized core-shell silica coated Fe_3O_4 nanomaterials for magnetic extraction of Pb(II) and Cr(VI) ions

Tehreema Nawaz,[1] Sonia Zulfiqar,[2] Muhammad Ilyas Sarwar [3,*] and Mudassir Iqbal[1]

[1]*Department of Chemistry, School of Natural Sciences, National University of Sciences and Technology, H-12, Islamabad, 44000, Pakistan.*

[2]*Department of Chemistry, School of Sciences & Engineering, The American University in Cairo, New Cairo, 11835, Egypt*

[3]*Department of Chemistry, Quaid-i-Azam University, Islamabad, 45320, Pakistan.*

* Corresponding author. M.I. Sarwar, Tel.: +92-51-90642132, Fax: +92-51-90642241,

E-mail address: ilyassarwar@hotmail.com ; ilyas@qau.edu.pk

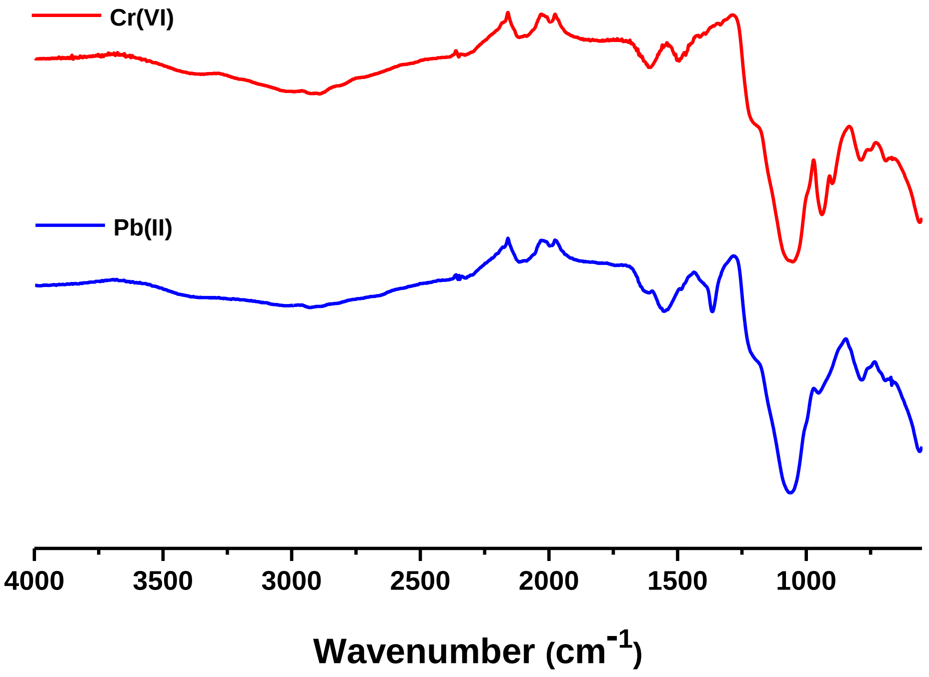

Fig. S1 FTIR spectra of Pb(II) and Cr(VI) after adsorption on FGA-1.

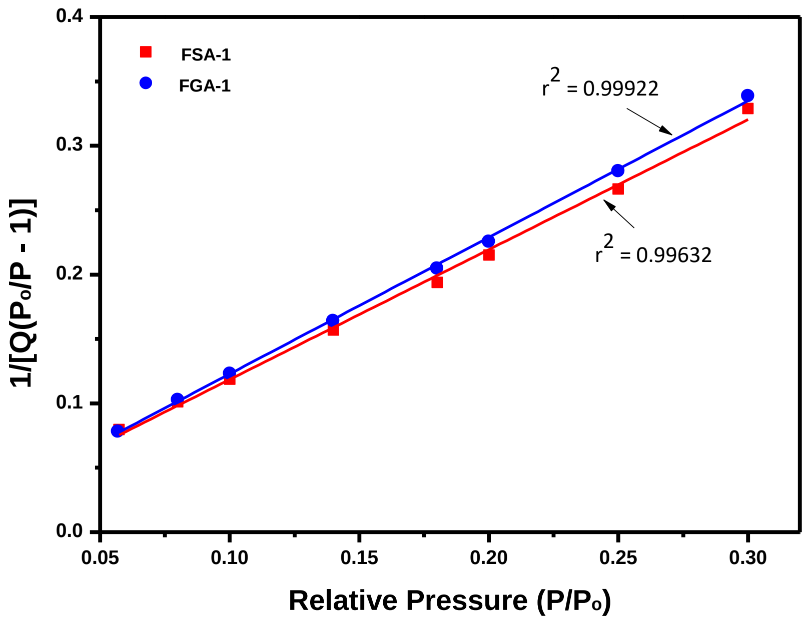

Fig. S2 BET linear plots of FSA-1 and FGA-1 from N_2 isotherms at 77K.

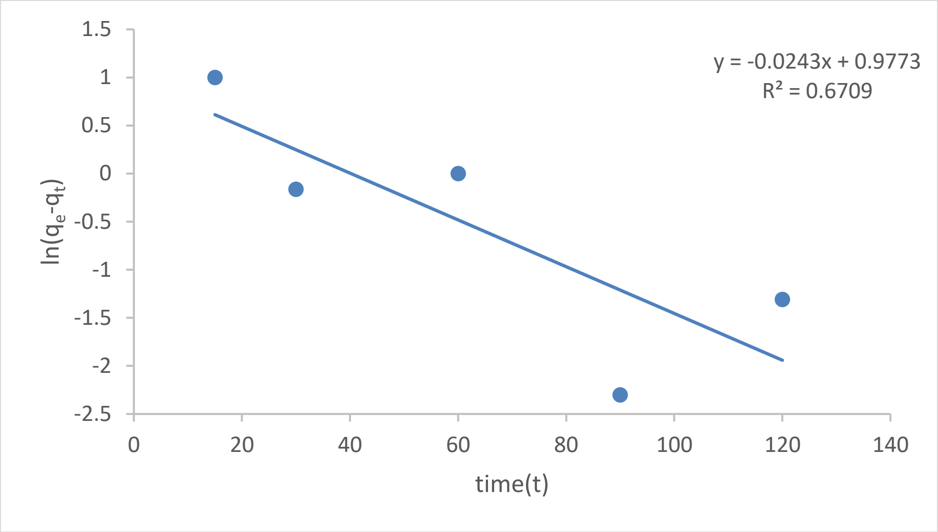

Fig. S3 Pseudo-first-order kinetics for the adsorption of Pb(II) by acid functionalized nanoparticles

Table S1 Pseudo-first order kinetic parameters for the adsorption of Pb(II) by acid functionalized nanoparticles

**Slope**	**Intercept**	**q_e**	**K_1**	**R^2**
-0.024	0.977	2.66	-0.024	0.671

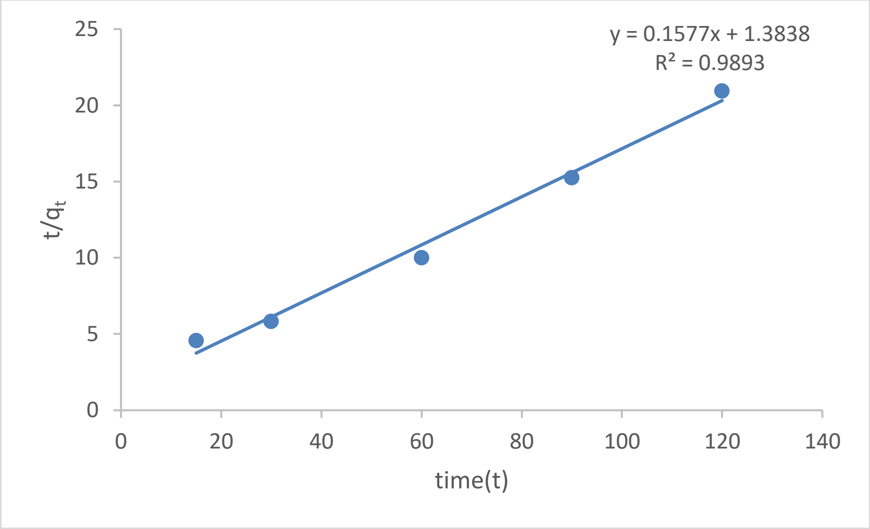

Fig. S4 Pseudo-second-order kinetics for the adsorption of Pb(II) by acid functionalized nanoparticles

Table S2 Pseudo-second-order kinetic parameters for the adsorption of Pb(II) by acid functionalized nanoparticles

Slope	Intercept	Q_e	K_2	R^2	$q_{e(Theoretical)}$	$q_{e\ (Experimental)}$
0.158	1.384	6.341	0.018	0.99	7.14	7.54

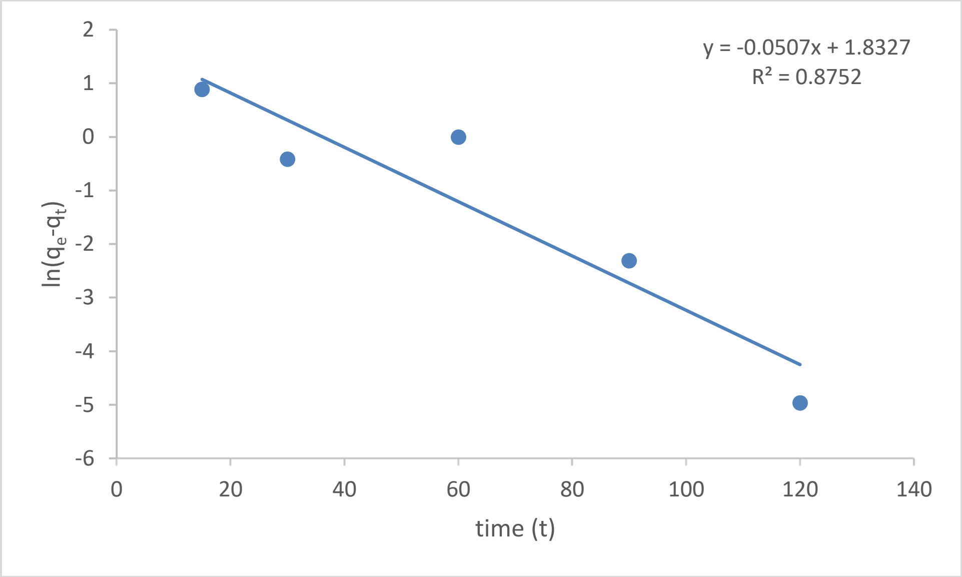

Fig. S5 Pseudo-first-order kinetics for the adsorption of Cr(VI) by acid functionalized nanoparticles

Table S3 Pseudo-first order kinetic parameters for the adsorption of Cr(VI) by acid functionalized nanoparticles

Slope	Intercept	q_e	K_1	R^2
-0.051	1.833	6.251	-0.051	0.87

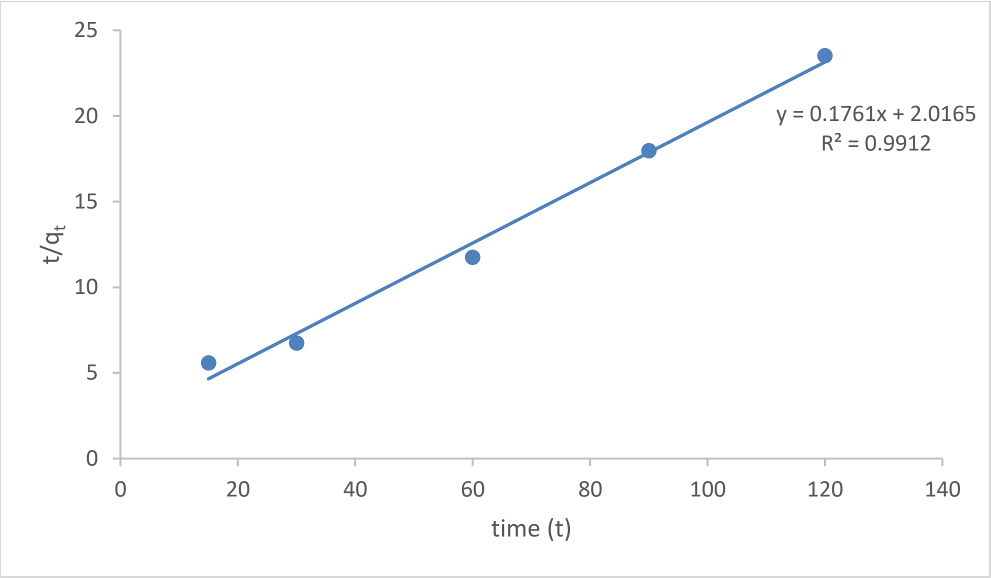

Fig. S6 Pseudo-second-order kinetics for the adsorption of Cr(VI) by acid functionalized nanoparticles

Table S4 Pseudo-second-order kinetic parameters for the adsorption of Cr(VI) by acid functionalized nanoparticles

**Slope**	**Intercept**	**Q_e**	**K_2**	**R^2**	**$q_{e(Theoretical)}$**	**q_{e} (Experimental)**
0.176	2.017	5.678	0.015	0.99	7.90	8.54

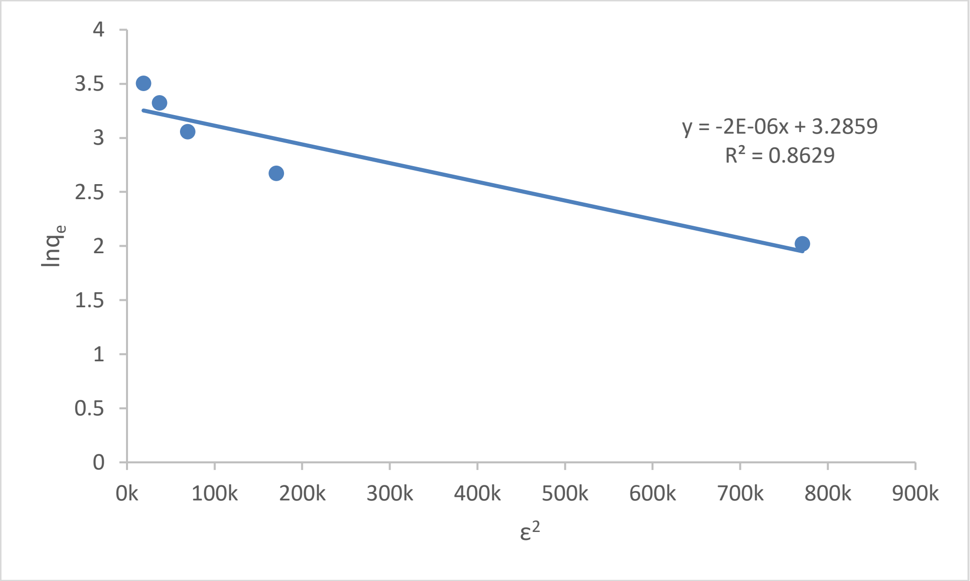

Fig. S7 Dubinin-Raduchkevick adsorption isotherm for Pb(II) ions by acid functionalized nanoparticles

Table S5 D-R isotherm parameters

Slope	Intercept	B (mol^2/KJ^2)	lnq_m	q_m (mg/g)	E (KJ/mol)
$-2*10^{-6}$	3.286	$-2*10^{-6}$	3.286	26.73	0.5

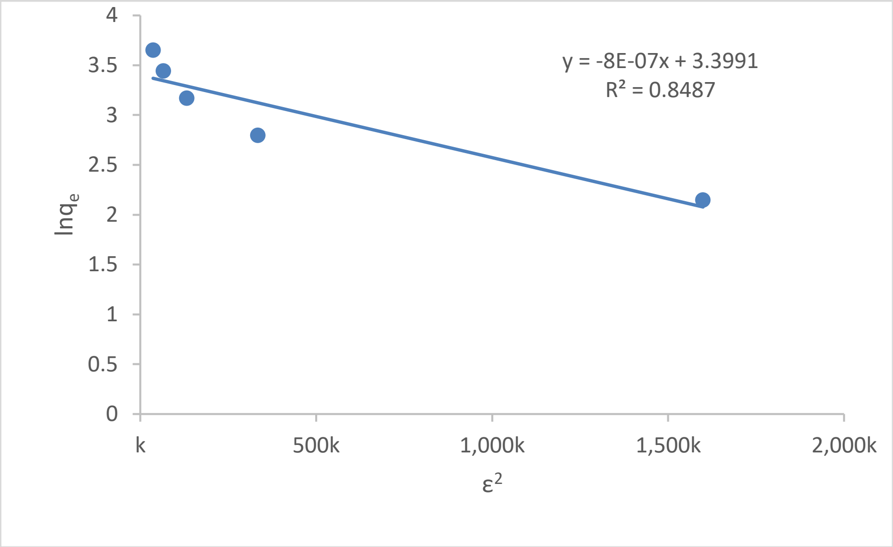

Fig. S8 Dubinin-Raduchkevick adsorption isotherm for Cr(VI) ions by acid functionalized nanoparticles

Table S6 D-R isotherm parameters

Slope	Intercept	B (mol^2/KJ^2)	lnq_m	q_m (mg/g)	E (KJ/mol)
$-8*10^{-7}$	3.399	$-8*10^{-7}$	3.399	29.94	0.79
